# Deconstructing Cancer Patient Information Seeking in a Consumer Health Library Toward Developing a Virtual Information Consult for Cancer Patients and Their Caregivers: A Qualitative, Instrumental Case Study

**DOI:** 10.2196/cancer.6933

**Published:** 2017-05-24

**Authors:** Janet Papadakos, Aileen Trang, Alaina B Cyr, Nazek Abdelmutti, Meredith E Giuliani, Michelle Snow, Tara McCurdie, Menaka Pulandiran, Sara Urowitz, David Wiljer

**Affiliations:** ^1^ Cancer Education Program Princess Margaret Cancer Centre University Health Network Toronto, ON Canada; ^2^ Radiation Medicine Program Princess Margaret Cancer Centre University Health Network Toronto, ON Canada; ^3^ Department of Radiation Oncology University of Toronto Toronto, ON Canada; ^4^ Health Care Human Factors Techna Institute University Health Network Toronto, ON Canada; ^5^ Department of Supportive Care Princess Margaret Cancer Centre University Health Network Toronto, ON Canada; ^6^ Canadian Cancer Research Alliance Toronto, ON Canada; ^7^ Education, Technology & Innovation University Health Network Toronto, ON Canada; ^8^ Department of Psychiatry University of Toronto Toronto, ON Canada; ^9^ Institute of Health Policy, Management and Evaluation University of Toronto Toronto, ON Canada

**Keywords:** patient education, information-seeking behavior, health literacy, Internet, consumer health information

## Abstract

**Background:**

Cancer patients and their caregivers want information about their disease and are interested in finding health information online. Despite the abundance of cancer information online, it is often fragmented, its quality is highly variable, and it can be difficult to navigate without expert-level knowledge of the cancer system. The Patient & Family Library at the Princess Margaret Cancer Centre offers a broad collection of high-quality cancer health information and staff are available to help patrons refine their questions and explore information needs that they may not have considered.

**Objective:**

The purpose of this research study was to deconstruct patrons’ information-seeking behaviors in the library to assess the feasibility of replicating the services provided in the library through a Web app, extending the service beyond the walls of the cancer centre. The specific aims of this research were to understand (1) how patrons approach information seeking in the library (interface design), (2) how patrons communicate their informational needs (information categorization and metadata requirements), and (3) what resources are provided to address the patrons’ information needs (collection development).

**Methods:**

We employed a qualitative, instrumental case study to deconstruct patrons’ health information-seeking behavior. The study population included patients, the librarian, and library volunteers. Ethnographic observation was conducted at the library over 3 days and key informant interviews with library staff were conducted to address the first aim. A closed card-sorting activity was conducted to address the second aim and the library shift logs and Search Request Forms (SRFs) were reviewed to address the third aim.

**Results:**

A total of 55 interactions were recorded during the ethnographic observation and nine semistructured interviews were conducted during the key informant interviews. Seven library patron personas were identified: (1) Newbie, (2) Seasoned, (3) Direct, (4) Window Shopper, (5) Collector, (6) Information Seeker, and (7) Distressed. A total of 83 participants completed the closed card-sorting exercise. The participants’ conceptual clusters within the similarity matrix overlapped with the groupings created by the librarian, with a few differences. A total of 161 entries in the library shift log and 65 SRFs were analyzed to determine what resources were given to patrons. Most resources that patrons received were available online (61%), although almost half of these required special access (47%).

**Conclusions:**

The study findings suggest it is possible to replicate library functions in a Web app with a few exceptions that cannot be replicated online. These elements include access to journal articles or other content behind paywalls and the librarian’s ability to encourage further discussion through empathy and active listening. Discussion with the librarian could serve to refine and predict needs through observing information seekers and to provide immediate connection to spiritual care and psychosocial support for patrons in distress.

## Introduction

It is well established that patients want and need information pertaining to their cancer [1-3]. Benefits of having their information needs met include having a better understanding of their disease, improved ability to cope, reduced anxiety, and satisfaction with treatment choices and health outcomes [4-9]. However, many patients do not know what information they may need because they are not familiar with medical protocols or cancer treatment. Furthermore, studies have shown that the amount and type of information desired varies depending on personal preference and phase in the cancer trajectory [7,9-11].

While health care providers are patients’ preferred source for health information [4,9], the Internet is becoming a prominent source for health-related information [8,9,12-16]. Finding and accessing good-quality health information online remains a problem for many patients [17,18]. Some challenges reported in the literature include the following: large amounts of health information available online can cause information overload [19,20], patients do not feel well equipped to assess the quality and credibility of information found online [7,19], the language level used is often far above the recommended grade 6 level for consumer health information [18,21], and there is a discordance between the design of most health information websites and the approach to searches that health information seekers employ [22,23]. A 2015 survey of patients with chronic health conditions found that approximately half of participants had difficulty finding health information, had low confidence in their searching abilities, and wanted support or guidance to find health information on the Internet [24,25]. Previous studies have also found that patients prefer health information websites that are recommended or created by trusted hospitals or health institutions [4,7,26].

The Princess Margaret Cancer Centre is the largest cancer centre in Canada and is located in downtown Toronto, Ontario. The Patient & Family Library (PFL) at the Princess Margaret Cancer Centre offers a broad collection of high-quality cancer health information. As part of their role, the librarian conducts *information consults* to help people find resources, understand and refine their information needs, and explore other possible information and support resources. Unfortunately, information consults can only be provided within the library. The ultimate aim of this study is to collect information to extend this service beyond the walls of the cancer centre by replicating the information consult online in the form of a *virtual information consult* (VIC). To do so requires a better understanding of the process behind the information consults and a better understanding of patient health information-seeking behaviors. A research study was conducted to deconstruct the information consults, specifically to understand the following: how patrons approach information seeking in the PFL, how patrons communicate their information needs, and what types of resources are provided to address the patron’s information needs. Based on these findings, the research team has assessed the feasibility of building an online format. Each study aim from above will also help inform, respectively, the interface design, the language and information categorization, and the online resource collection.

The PFL assists patrons, often patients or their caregivers, in finding health information they may need throughout the cancer care trajectory. The PFL houses a variety of health-information materials in a variety of formats—pamphlets, lendable books, DVDs, and CDs—that patrons can browse on their own or with the assistance of a trained library staff member. Brief interactions between staff and patrons—a patron asks a simple question resulting in a straightforward response—happen quite frequently and are manually tracked in the PFL shift log. When a patron’s information needs require a more in-depth discussion, the librarian may conduct an information consult with patrons to help them understand and refine their information needs, find resources to meet their needs, and explore other topics that the patron may not have considered. These information consults usually consist of a 10-15-minute conversation between the patron and the librarian and are tracked in the PFL shift log. Complex information requests that are not readily addressed by the PFL’s resource collection can be submitted to the librarian using a Search Request Form (SRF). To respond to an SRF query, the librarian searches for current and credible resources online and, when needed, scientific literature to address the patron’s request, and creates a tailored information package including a cover letter. Patrons can pick up the package or receive it by mail or through email. These services are only available to patrons who visit the PFL.

## Methods

### Overview

A qualitative, instrumental case study was employed to deconstruct and examine how patrons seek and find health information in the PFL. Qualitative case study methodologies employ a variety of qualitative methods to answer “how” and “why” questions about complex phenomena within the context they occur [27,28].

### Study Sample

The study sample was comprised of target end users of VIC. A convenience sample of patients (from initial diagnosis to long-term follow-up), caregivers, and PFL staff from the Princess Margaret Cancer Centre were recruited to participate in the different phases of this research study, described below. Potential participants were excluded if they were under the age of 18, were not fluent in English, or unable to provide informed consent.

### Data Collection and Analysis

#### How Patrons Approach Information Seeking in the Patient & Family Library (Interface Design)

##### Ethnographic Observation

Ethnographic observation was conducted to gain a better understanding of how library patrons get from point of entry to finding/receiving resources that address their information needs. The ethnographic observation was conducted in collaboration with the University Health Network’s Healthcare Human Factors (HHF) group, a diverse team of human factors specialists who are trained in human factor principles, design, and evaluation, including usability evaluations and ethnographic studies on clinical users and their environments. Two members of the HHF group independently observed the interactions between library staff and patrons visiting the PFL over the course of 3 days. During each interaction, the observers recorded details of the visitor’s movements throughout the PFL, including any conversation the visiting patron may have had with PFL staff. The observers did not directly interact with visiting patrons. Following each interaction, the observers conducted a short debrief with the librarian to ensure that the nature of the interaction was accurately recorded. The observers conducted thematic analyses of the recorded interactions and personas emerged. The observers discussed the themes at length and once satisfied that they had captured the various approaches to information seeking and had categorized them appropriately, presented their findings to the study team to discuss and refine the personas. The study team supported the findings and only two changes were made. The first change was to collapse two personas into one, as there was much overlap between the two; the second change was to refine the names assigned to the personas in an effort to make them more descriptive.

##### Key Informant Interviews

Semistructured interviews were conducted in person with the PFL library staff to complement the findings from the ethnographic observation. Informants were recruited through an email describing the study and inviting them to participate in the interview. During the 60-minute interview, informants were asked open-ended questions intended to capture the kinds of discussions that occurred between the library staff and patrons, with a particular focus on how the library staff assisted patrons in identifying and/or expressing their information needs, and what resources were provided. Interviews were audio recorded and transcribed, and a thematic analysis was performed on the transcripts.

#### How Patrons Communicate Information Needs (Information Categorization and Metadata Requirements)

A card-sorting exercise was performed. Card sorting is a technique that is often used in Web development to better understand users’ mental models by examining how individuals view items as relating to each other and how they group items into categories [29]. A closed card-sorting exercise was used to determine how similar the conceptual categories patients used to group information were to the conceptual categories used by the librarian.

The titles and descriptions of 50 resources from the PFL catalog were randomly selected to be used during the card-sorting exercise. Each card included the full title of the resource and a brief description—one or two sentences—to describe the type of information contained in each resource. The format (ie, book, video, website, etc) was not included on the card. The subject headers under which each resource was cataloged in the PFL were used as the categories into which participants could choose to sort the cards.

Participants for the card-sorting exercise were recruited by staff in the PFL and passively through recruitment posters distributed throughout the hospital. An invitation to participate in this research study was also sent to an electronic distribution list of patients and caregivers who had previously consented to be sent invitations to participate in research.

OptimalSort software (Optimal Workshop) was used to administer the card-sorting exercise, facilitate data collection, and record responses. The OptimalSort interface displayed the names of the 50 resources on cards to participants. As with typical closed card-sorting exercises, the participants were instructed to sort the 50 cards into the categories provided using drag and drop. Some examples of titles of resources selected for card sorting included *How to use your feeding tube*, *Food safety for patients with weakened immune systems*, and *Introduction to radiation therapy*.

The same 50 cards were also sorted by the librarian into the PFL categories to provide a basis to compare patient conceptual groupings to those used in the PFL. A similarity matrix was created with the aggregate participant card-sorting results; it was then reordered using the librarian’s groupings to allow for easier comparison. If participants’ conceptual categories were a match with the librarian’s, we expected to see clusters of agreement along the diagonal axis, corresponding within each category.

#### What Resources Are Provided to Patients and Caregivers (Collection Development)

Document analysis, including a retrospective review of 1 month of the most recent PFL shift logs and 5 months of the most recently submitted SRFs, was conducted to provide insight into the information resources given to patrons and the types of topics requested. An additional review of these information resources was conducted to determine how many were available online. A descriptive analysis was performed on the quantitative data and a thematic and keyword analysis was performed on the qualitative data.

## Results

### How Patients and Caregivers Seek Information (Interface Design)

A total of 55 interactions were recorded during the ethnographic observation and nine semistructured interviews were conducted during the key informant interviews. A total of seven library patron personas were identified by the HHF researchers based on the ethnographic observation data. Personas emerged based on a combination of familiarity with the PFL, motivation for visit (where available), and specific information-seeking behavior. The personas were further supported when cross-referenced with the data extracted from the key informant interviews and shared with the study team. Patrons transition between different personas depending on their experience and information needs at different times. The personas identified included the following: (1) Newbie, (2) Seasoned, (3) Direct, (4) Window Shopper, (5) Collector, (6) Information Seeker, and (7) Distressed. Once the personas were established, the study team discussed Web apps that could be used to serve the needs of each type of persona. Descriptions of personas, information-seeking considerations for each persona, and the corresponding Web app recommendations are shown in Table 1.

**Table 1:** Description of personas, information-seeking considerations, and Web app recommendations.

Persona	Description	Information-seeking considerations	Corresponding Web app recommendation
Newbie	New visitor to the PFL^a^, looking for general information, possibly feeling overwhelmed, and not yet aware of the ample services and information available to them	Exploratory search strategy	Results filtering Topic-based menu options Support/tips section for using Web app Contact-us option
Seasoned	Familiar with the library and what it offers; require little assistance from the library staff and often come in looking for a specific resource	Focused search strategy	Search bar (with fuzzy search)
Window Shopper	Explore pamphlet racks and the front display table; unlikely to interact with the library staff and some may not enter the PFL	Exploratory search strategy	Card display with featured items enabling quick information scanning A-Z list of resources
Direct	Knows exactly what they are looking for, often coming in with a specific resource title request or a recommendation from a clinician; less likely to explore the library	Focused search strategy	Search bar (with fuzzy search) Ability to download and save resources
Collector	Some familiarity with the PFL and comes in to find information on a specific topic; collect as much as they can on that topic and do not seek anything further	Exploratory search strategy	Topic-based menu options Ability to download and save resources
Information Seeker	Engages in an information consult with the librarian and seeks as much information as possible; typically leaves the PFL with a comprehensive set of resources	Focused search strategy	Topic-based menu options Contact-us option
Distressed	Visibly upset and preoccupied; may seek either general or specific information, but not so much information that it overwhelms them; may also need someone to talk to such as the library staff, spiritual care, or psychosocial support	Varied	Clear social support section (including audio-based mindfulness exercises and clear list of support services) Neutral color palette and single font type

^a^PFL: Patient & Family Library.

### How Patrons Communicate Information Needs (Information Categorization and Metadata Requirements)

A total of 83 participants completed the closed card-sorting exercise. To aid with identifying categories, card pairings that were paired by 27 or more participants were color coded. These highlighted cells were then compared to the librarian’s categories to determine if cards were sorted similarly between the participants and the librarian.

The participants’ conceptual categories within the similarity matrix overlapped with the conceptual categories created by the librarian, with only a few differences. Participant categorization of 10 of the 50 cards did not agree with the categorization used by the librarian, as seen in Textbox 1. In these few cases, the librarian’s conceptual categories were broader in comparison to those of the participants. For example, the librarian categorized the resource *How to use your feeding tube* under the category *Self-management*, while many participants categorized it under *Eating*. The librarian also categorized several resources that related to coping with cancer under the broad category *Coping*, while many participants assigned multiple categories to these resources, including *Healing*, *Testimonials*, and *Help*. Examples of the conceptual categories of participants that matched with those of the librarian include the following: *Side Effects*, *Prevention*, and *Treatments*.

**The 10 resources that did not match in the participants’ and librarian’s conceptual categorizations.**

Card titles

1. How to Use Your Feeding Tube

2. Relaxation Therapy

3. Caregiver Stress

4. Anti-Cancer

5. Living with Advanced Cancer

6. Spiritual Care

7. Coping With Cancer—Psychosocial Oncology

8. The Healing Journey

9. Where's Mom's Hair?

10. When a Parent Has Cancer

### What Resources Are Provided to Patients and Caregivers (Resource Collection Development Policy)

A total of 161 entries in the PFL shift log and 65 SRFs were analyzed to determine what resources were given to PFL patrons. The PFL shift logs recorded simple queries on topics ranging from health information and support to directions in and around the hospital. The most common types of resources distributed to address these queries were pamphlets (distributed in 93% of the queries), books (distributed in 16% of these queries), and e-books (distributed in 2% of these queries).

The queries recorded in the SRFs were for cancer information (33%), specific treatment information (32%), clinical trials and/or research articles (20%), and rare cancer information (15%). The most common types of resources provided to address SRFs were credible cancer websites (62%), followed by research articles and clinical trial webpages (48%), which require special access to use. Fewer queries required books, pamphlets and brochures (38%), classes, events and support groups (23%), or contact information of a health professional (9%). Most search request resources were available online (61%); however, 47% of these resources were not available to the general public.

## Discussion

### Principal Findings

After deconstructing how patrons seek information in the PFL, examining the mental models they use to categorize health information, and reviewing the types of resources provided by PFL staff, we determined it was feasible to develop an online complementary tool that can assist cancer patients and caregivers in finding high-quality cancer health information. However, it is not possible to replicate all of the PFL functions in entirety.

Seven patron personas were observed in the PFL that underline the different ways that patients and caregivers approach information seeking, complementing previous research that found that users seek health information by using a focused or an exploratory approach [23,30,31]. Focused searchers use specific keywords and refine their search until they find the answer they are looking for [32,33]. The Direct, Seasoned, and Information Seeker personas identified in our study appear to use focused search strategies to find health information. Exploratory searchers are less confident about what they are looking for [23,31,34] and may explore a variety of information during their search [30,32]. The Newbie, Window Shopper, and Collector personas identified in the study appear to use exploratory search strategies to find health information.

The needs of six personas—Newbie, Direct, Seasoned, Collector, Window Shopper, and Information Seeker—may be met by including certain features in the VIC interface design. Conducting a search is the most common starting point for consumers seeking health information [13,21,35,36]. A search box would likely be sufficient for the Direct and Seasoned personas who approach information seeking with confidence, though a “fuzzy search” function should be added to allow for misspelling. The Newbie persona may begin with a search, but find they need assistance narrowing down their results, a strategy employed by many health information seekers called *orienteering* [37]. The search results page could contain a number of filters to guide users in refining the results to better meet their information needs. The Window Shopper persona is unlikely to conduct an in-depth search using the search bar, but there is evidence that search engine advertisements can provide teachable moments for users who rarely go beyond the first page of search results [38]. Thus, resource “advertisements” on the VIC landing page may provide Window Shoppers with sufficient information to meet their needs. The Collector and Information Seeker personas seek out everything available on a specific topic. To aid in this information-seeking behavior, topic-based menu options should be constantly present on the interface to encourage these personas to dig deeper within the search [39]. Additionally, subject headings could be displayed alongside resources returned in a search query to assist the Collector and Information Seeker personas in determining all topics related to their query. Displaying the subject headings along with the resources may also assist the Newbie with orienteering.

Addressing the needs of the Distressed persona is more challenging as technology cannot replace human interaction and immediate connection to psychosocial support that is possible in the PFL. No studies have examined the effectiveness of online self-help interventions for cancer-related distress; however, there is significant evidence to suggest mindfulness-based stress reduction programs are effective interventions for managing cancer-related distress [40-44]. These interventions are typically in-person classes that meet over the course of several weeks. One reported study assessed the feasibility of conducting a mindfulness-based stress reduction program for cancer patients online and found the effects were similar to in-person programs [45,46]. We propose including an audio-based guided mindfulness exercise on the VIC interface to provide immediate support for the Distressed persona. Advertised alongside this mindfulness exercise, we can include a list of psychological and spiritual support services, linking to basic service information and details on how a patient can get a referral. Future research could examine the effectiveness of our approach in reducing distress in the moment.

When examining how patrons organize health information, it was determined that the mental model they used was similar to that used by librarians in the PFL, and thus categorizing information using the subject headings used in the PFL would not hinder users’ ability to find information. The conceptual clusters created by the participants were not a precise match for those used in the PFL; however, the differences may be attributed to the data collection method. The protocol limited the categorization of each resource to precisely one category, requiring participants to use their judgment to determine best fit. In practice, the PFL catalog associates multiple subject headings to each resource. Differences in conceptual clusters may also be attributed to differing familiarity of the resources included in the card-sort activity. The librarian had in-depth knowledge of each resource in the activity while participants’ knowledge was limited to the title and brief description provided on the card.

In examining the resources provided to patrons by PFL staff, most were related to cancer topics including information on rare types, specific treatments, and clinical trials, with more than half of these resources available online. Previously, the research team determined that the collection development policy for the PFL should focus on relevance to patron population, credibility, currency, and accessibility of the content and its format [47]. The same criteria could be applied to an online catalog, though the scope of *accessibility* would need to broaden to include digital accessibility. In many cases, the information provided in the PFL is already accessible online, though not within a single Web app. All pamphlets created by Princess Margaret Patient Education are available on the Princess Margaret Cancer Centre website, as are details about support services, specialized programs, classes, and active clinical trials that take place at Princess Margaret Cancer Centre. Digital complements of information from external organizations are often available online as webpages or downloadable PDFs. An online public access catalog, also published on the Princess Margaret Cancer Centre website, allows users to browse the PFL lendable materials, though they must visit the PFL to pick up the resource. Digital accessibility becomes a challenge with respect to restricted-access websites, as it is not technically feasible to support public access to content behind paywalls or restricted areas. While it is not feasible to catalog these particular types of resources in VIC, the contact information of the PFL could be included to help facilitate patient access to this content or provide support should a search be too complex to be handled with VIC.

Since the completion of the study, an interface prototype of VIC has been built, tested with users, and refined through several iterations. Next steps involve collaborating with Web developers to build the VIC database and user interface. Future studies will involve evaluating the VIC interface.

### Conclusions

The purpose of this study was to deconstruct the health information-seeking behavior of patrons of the PFL to determine the feasibility of building a Web app that could assist patients and caregivers with finding high-quality cancer health information. Through observing daily operations in the PFL, seven patron personas were identified that describe how patients and caregivers approach information seeking. The subject headings used by the librarian were compared to groupings used by patrons and we found that they were a close match. The majority of resources given to patrons were available online.

The study findings suggest it is possible to replicate the services of the PFL in a Web app, with a few exceptions. The elements that cannot be replicated online include access to journal articles or other content behind paywalls and the librarian’s ability to encourage further discussion through empathy and active listening. Further discussion with the librarian could serve to refine and predict needs through observing information seekers and provide immediate connection to spiritual care and psychosocial support for patrons in distress.

## Abbreviations

HHF: Healthcare Human Factors
PFL: Patient & Family Library
SRF: Search Request Form
VIC: virtual information consult

## References

[1] Chen X, Siu LL (2001). Impact of the media and the Internet on oncology: Survey of cancer patients and oncologists in Canada. J Clin Oncol.

[2] Hesse BW, Arora NK, Burke BE, Finney Rutten LJ (2008). Information support for cancer survivors. Cancer.

[3] Roach AR, Lykins EL, Gochett CG, Brechting EH, Graue LO, Andrykowski MA (2009). Differences in cancer information-seeking behavior, preferences, and awareness between cancer survivors and healthy controls: A national, population-based survey. J Cancer Educ.

[4] Eysenbach G (2003). The impact of the Internet on cancer outcomes. CA Cancer J Clin.

[5] Jefford M, Tattersall MH (2002). Informing and involving cancer patients in their own care. Lancet Oncol.

[6] Klemenc-Ketis Z, Kersnik J (2013). Seeking health advice on the Internet in patients with health problems: A cross-sectional population study in Slovenia. Inform Health Soc Care.

[7] Mayer DK, Terrin NC, Kreps GL, Menon U, McCance K, Parsons SK, Mooney KH (2007). Cancer survivors information seeking behaviors: A comparison of survivors who do and do not seek information about cancer. Patient Educ Couns.

[8] Rozmovits L, Ziebland S (2004). What do patients with prostate or breast cancer want from an Internet site? A qualitative study of information needs. Patient Educ Couns.

[9] Rutten LJ, Arora NK, Bakos AD, Aziz N, Rowland J (2005). Information needs and sources of information among cancer patients: A systematic review of research (1980-2003). Patient Educ Couns.

[10] Alzougool B, Chang S, Gray K (2013). Information Research.

[11] James N, Daniels H, Rahman R, McConkey C, Derry J, Young A (2007). A study of information seeking by cancer patients and their carers. Clin Oncol (R Coll Radiol).

[12] Bundorf MK, Wagner TH, Singer SJ, Baker LC (2006). Who searches the Internet for health information?. Health Serv Res.

[13] Fox S (2013). After Dr Google: Peer-to-peer health care. Pediatrics.

[14] Oh YS, Cho Y (2015). Examining the relationships between resources and online health information seeking among patients with chronic diseases and healthy people. Soc Work Health Care.

[15] Tustin N (2010). The role of patient satisfaction in online health information seeking. J Health Commun.

[16] Wagner TH, Baker LC, Bundorf MK, Singer S (2004). Use of the Internet for health information by the chronically ill. Prev Chronic Dis.

[17] Burns P, Jones SC, Iverson DC, Caputi P (2013). Where do older Australians receive their health information? Health information sources and their perceived reliability. J Nurs Educ Pract.

[18] Sium A, Giuliani M, Papadakos J (2015). The persistence of the pamphlet: On the continued relevance of the health information pamphlet in the digital age. J Cancer Educ.

[19] Cline RJ, Haynes KM (2001). Consumer health information seeking on the Internet: The state of the art. Health Educ Res.

[20] Flanagin AJ, Metzger MJ (2011). From Encyclopaedia Britannica to Wikipedia. Inf Commun Soc.

[21] Lee K, Hoti K, Hughes JD, Emmerton L (2014). Dr Google and the consumer: A qualitative study exploring the navigational needs and online health information-seeking behaviors of consumers with chronic health conditions. J Med Internet Res.

[22] Pang P, Verspoor K, Pearce J, Chang S (2015). Better health explorer: Designing for health information seekers. Proceedings of the Annual Meeting of the Australian Special Interest Group for Computer Human Interaction.

[23] Pang PC, Chang S, Verspoor K, Pearce J (2016). Designing health websites based on users' Web-based information-seeking behaviors: A mixed-method observational study. J Med Internet Res.

[24] Lee K, Hoti K, Hughes JD, Emmerton LM (2015). Consumer use of “Dr Google”: A survey on health information-seeking behaviors and navigational needs. J Med Internet Res.

[25] Lee YJ, Boden-Albala B, Jia H, Wilcox A, Bakken S (2015). The association between online health information-seeking behaviors and health behaviors among Hispanics in New York City: A community-based cross-sectional study. J Med Internet Res.

[26] van de Poll-Franse LV, van Eenbergen MC (2008). Internet use by cancer survivors: Current use and future wishes. Support Care Cancer.

[27] Baxter P, Jack S (2008). The Qualitative Report.

[28] Yin RK (2009). Case Study Research: Design and Methods. 4th edition.

[29] Capra MG (2005). Factor analysis of card sort data: An alternative to hierarchical cluster analysis. Proceedings of the Human Factors and Ergonomics Society Annual Meeting.

[30] Marchionini G (2006). Exploratory search: From finding to understanding. Commun ACM.

[31] White RW, Roth RA, Marchionini G (2009). Exploratory Search: Beyond the Query-Response Paradigm.

[32] Pang PC, Chang S, Verspoor K, Pearce J (2016). Designing health websites based on users' Web-based information-seeking behaviors: A mixed-method observational study. J Med Internet Res.

[33] Pang PC, Chang S, Pearce J, Verspoor K (2014). Online health information seeking behaviour: Understanding different search approaches. Proceedings of the 18th Pacific Asia Conference on Information Systems.

[34] Pearce J, Chang S, Alzougool B, Kennedy G, Ainley M, Rodrigues S (2011). Search or explore: Do you know what you're looking for?. Proceedings of the 23rd Australian Computer-Human Interaction Conference.

[35] Lee K, Hoti K, Hughes JD, Emmerton LM (2014). Interventions to assist health consumers to find reliable online health information: A comprehensive review. PLoS One.

[36] Tian H, Brimmer DJ, Lin JS, Tumpey AJ, Reeves WC (2009). Web usage data as a means of evaluating public health messaging and outreach. J Med Internet Res.

[37] Ossebaard HC, Seydel ER, van Gemert-Pijnen L (2012). Online usability and patients with long-term conditions: A mixed-methods approach. Int J Med Inform.

[38] Cooper CP, Gelb CA, Vaughn AN, Smuland J, Hughes AG, Hawkins NA (2015). Directing the public to evidence-based online content. J Am Med Inform Assoc.

[39] Danielson DR (2002). Web navigation and the behavioral effects of constantly visible site maps. Interact Comput.

[40] Carlson LE (2016). Mindfulness-based interventions for coping with cancer. Ann N Y Acad Sci.

[41] Cramer H, Lauche R, Paul A, Dobos G (2012). Mindfulness-based stress reduction for breast cancer: A systematic review and meta-analysis. Curr Oncol.

[42] Musial F, Büssing A, Heusser P, Choi K, Ostermann T (2011). Mindfulness-based stress reduction for integrative cancer care: A summary of evidence. Forsch Komplementmed.

[43] Piet J, Würtzen H, Zachariae R (2012). The effect of mindfulness-based therapy on symptoms of anxiety and depression in adult cancer patients and survivors: A systematic review and meta-analysis. J Consult Clin Psychol.

[44] Smith JE, Richardson J, Hoffman C, Pilkington K (2005). Mindfulness-based stress reduction as supportive therapy in cancer care: Systematic review. J Adv Nurs.

[45] Zernicke KA, Campbell TS, Speca M, McCabe-Ruff K, Flowers S, Carlson LE (2014). A randomized wait-list controlled trial of feasibility and efficacy of an online mindfulness-based cancer recovery program: The eTherapy for cancer applying mindfulness trial. Psychosom Med.

[46] Zernicke KA, Campbell TS, Speca M, McCabe-Ruff K, Flowers S, Dirkse DA, Carlson LE (2013). The eCALM Trial-eTherapy for cancer appLying mindfulness: Online mindfulness-based cancer recovery program for underserved individuals living with cancer in Alberta: Protocol development for a randomized wait-list controlled clinical trial. BMC Complement Altern Med.

[47] Papadakos J, Trang A, Wiljer D, Cipolat MC, Cyr A, Friedman AJ, Mazzocut M, Snow M, Raivich V, Catton P (2014). What criteria do consumer health librarians use to develop library collections? A phenomenological study. J Med Libr Assoc.

